# Optimizing clinical risk stratification of localized prostate cancer

**DOI:** 10.1097/MOU.0000000000001294

**Published:** 2025-05-02

**Authors:** Vincent J. Gnanapragasam

**Affiliations:** a Department of Surgery, University of Cambridge; b Cambridge Prostate Cancer and Clinical Trials Group; c Cambridge University Hospitals, Urology, Cambridge, UK

**Keywords:** artificial intelligence, genomic classifiers, modern clinical classifiers, MRI, prognosis, prostate cancer

## Abstract

**Purpose of review:**

To review the current risk and prognostic stratification systems in localised prostate cancer. To explore some of the most promising adjuncts to clinical models and what the evidence has shown regarding their value.

**Recent findings:**

There are many new biomarker-based models seeking to improve, optimise or replace clinical models. There are promising data on the value of MRI, radiomics, genomic classifiers and most recently artificial intelligence tools in refining stratification. Despite the extensive literature however, there remains uncertainty on where in pathways they can provide the most benefit and whether a biomarker is most useful for prognosis or predictive use. Comparisons studies have also often overlooked the fact that clinical models have themselves evolved and the context of the baseline used in biomarker studies that have shown superiority have to be considered.

**Summary:**

For new biomarkers to be included in stratification models, well designed prospective clinical trials are needed. Until then, there needs to be caution in interpretation of their use for day-to-day decision making. It is critical that users balance any purported incremental value against the performance of the latest clinical classification and multivariate models especially as the latter are cost free and widely available.

## INTRODUCTION

Prostate cancer is a complex disease to manage because of its very variable clinical course. In many cases, a diagnosis of prostate cancer will not result in mortality or morbidity from the disease [1]. Hence, understanding the risks posed from a prostate cancer diagnosis is critical to its management [2–4]. All too often however, this understanding is poor amongst clinicians and leads to recommendations that can result in both over treatment and under treatment [5]. To aid these discussions, the use of risk and prognostic models have become essential in practice [2–4]. Until about 15 years ago, the main risk models used were based on the first iteration described by D’Amico nearly 30 years ago [6]. Crucially, the notion of “risk” was based on the chance of biochemical relapse thus assuming that treatment was an inevitable requirement in prostate cancer. It is now well established that many men especially with localised disease do need treatment and surveillance is an important management option [1–4]. Despite this, many national and international guidelines still recommend classifications that are iterations or derivatives of the now out-dated three-tier low, intermediate and high-risk model [3,4]. 

**Box 1:**
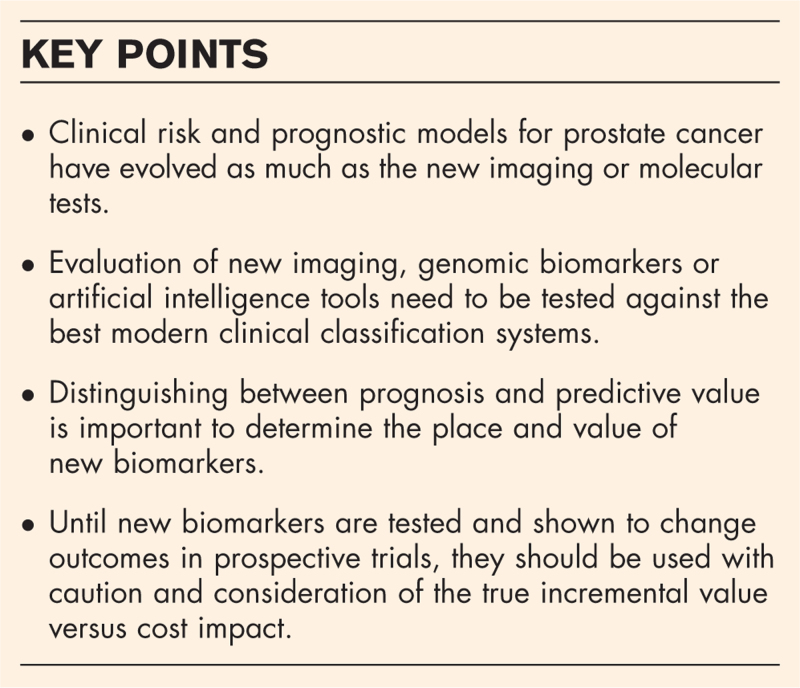
no caption available

## MODERN CLINICAL RISK AND PROGNOSIS MODELS

A key limitation of older risk models is that they were never tested or evaluated in their ability to predict oncological outcomes like metastasis, prostate cancer specific or overall mortality. The new generation of models however have been derived and calibrated against these outcomes and also included large numbers of men managed conservatively in their cohorts [7]. These represent significant improvements in terms of prognostic accuracy as has been shown in head-to-head comparison studies [8,9]. Modern models like the Cambridge Prognostic Groups, CAPRA score or STARCAP have shown accuracies of between 0.78 and 0.81 in predicting prostate cancer specific mortality outperforming the EAU/AUA and NCCN models [8,10,11]. Some, like the Cambridge Prognostic Groups, are now adopted into national recommendations (UK) replacing the three-tier risk model [2].

While representing significant improvements, these models remain “group classifiers” which do not allow for more individualised estimates of prognosis. This is a limitation in a disease primarily of older men in whom the competing risks that affect overall survival must be considered. In recent times, free to use individualised prognostic models that include competing risks have been developed for clinical use including the Predict Prostate and the MSKCC nomogram [7,12]. The Predict Prostate tool (https://prostate.predict.cam) provides individualized cancer-specific and overall long-term survival estimates in men with a new diagnosis of early prostate cancer. It has been developed, tested and validated in over 350 000 men from four countries and across multiple ethnicities [7,9]. In addition to the routinely available diagnostic clinical-pathological variables, the PREDICT Prostate tool also includes the impact of patient characteristics such as age and comorbidity and the likely benefit of treatment (radical prostatectomy or radiotherapy) versus surveillance on survival. It further provides (in a non-individualised readout) side effect impact that can occur up to 12 years after a decision for treatment or surveillance. Predict Prostate is endorsed and recommended by the UK NICE and EAU guidelines and is available in seven languages [2,3]. In head-to-head comparisons, Predict has been shown to outperform all other clinical classifications [9]. The MSKCC dynamic nomogram has been available for some time and was developed to help men make decisions about competing risks when considering surgery [11]. Unlike Predict Prostate, it does not provide a unified readout for cancer-specific and overall survival but has an option to determine likelihood of mortality from prostate cancer or other causes if a man receives no treatment [11]. These tools are in use thousands of times a year to help make individual decisions about the value of treatment especially in men of an older age or with early disease [13].

Clinical risk and prognostic models for prostate cancer have therefore evolved as much as new imaging or molecular tests. This fact however is often over looked when new studies are reported incorporating a new biomarker or test. Most studies have instead tested new biomarkers against (now considered) substandard clinical risk stratification models to show improved performance. Few have used multivariate competing risks models as baseline to test for incremental gain. Modern clinical models such as the CPG and Predict Prostate have also included men who were managed by active surveillance. In contrast, as discussed, the D’Amico-derived models like NCCN were never tested in such cohorts [10]. Against the backdrop of these uncertainties, we explore some of the most promising areas and what the evidence to date has shown regarding their value in addition to (cost-free) clinical models.

## IMAGING MODALITIES IN RISK PREDICTION

New imaging modalities in prostate cancer usually start with efforts to improve detection and staging and then studied for additive value in treatment prediction and prognosis. MRI has undoubtedly shown worth in improving the ability to detect cancer and perhaps more importantly who does not need investigation [14]. The data on treatment outcomes however are mixed. MRI has been linked to better prediction of extracapsular extension, biochemical relapse and prostate cancer mortality after radical prostatectomy specifically by its improved ability to detect T3 disease; in other words better pretreatment staging [15,16]. In contrast, other studies have found only a small advantage in adding MRI to currently used presurgical nomograms [17]. In the radiotherapy setting, pretreatment MRI parameters were associated with metastatic and mortality outcomes but again mainly due to its use as a staging tool (improved pretreatment detection of extra-prostatic extension, seminal vesicle invasion, presence of metastatic lymph nodes) [18].

A key question is if MRI lesion features (e.g. conspicuousness/Likert or PIRAD score) may add incremental value. The data so far are conflicting [19,20]. Stabile *et al.*[15] in a systematic review on the predictive role of the PI-RADS score found some evidence for a trend for better prediction of post radical prostatectomy biochemical relapse but not for other clinical endpoints. Interestingly, there was also no positive association between relapse and MRI tumour volume [15]. Others have similarly also reported associations between MRI features and biochemical relapse, but whether this has an impact on hard oncological outcomes such as prostate cancer specific mortality remains unknown [21,22]. Biochemical relapse itself is known to have variable definitions and associations with mortality [23]. Thus, in this context, MRI features are best considered potentially “predictive” of treatment failure rather than prognostic. Radiomics is a newer field which seeks to glean more complex data from imaging, to better phenotype tissue and different disease states. In prostate cancer, its role is being actively explored with most studies focusing on better cancer detection and classification, though no algorithms are yet in routine clinical use [24,25^▪^]. Promising data has now emerged on the use of radiomic features for predicting final prostatectomy histology, reducing rectal toxicity at radiotherapy and predicting progression on active surveillance [24,25^▪^,^26^].

To date therefore, there is little convincing evidence that MRI features provide independent value in prognosis prediction [15]. While it can improve stage allocations, this impact may be modest outside populations with a high prevalence of PSA testing and hence generally earlier stage presentations. Lophatananon *et al.*[27], for example, modelled the impact of MRI staging versus clinical staging in a newly diagnosed cohort of UK men and found stage shifts in only about 8% of cases. In terms of an individualised change in prognosis, the impact of MRI was minimal when modern group classifiers and prognostic tools were used [27]. Conversely, there are concerns that the use of MRI guides biopsies may instead artificially inflate risk and contribute to over-diagnosis and over-treatment [28]. Longer-term data will be required to see if and how prognostic models may need to be adjusted to account for MRI. At present, it remains doubtful that MRI measurements *per se* will add independent prognostic value. These same issues will no doubt arise when PSMA data matures over time.

## GENOMIC CLASSIFIERS

Genomic classifier profiling using extracted DNA/RNA from histological tissue have been explored in prostate cancer for over a decade and the number of tests appear to be increasing [29]. Current tests with the most data include Decipher, Oncotype Dx, Prolaris and the Genomic Prostate score [29,30^▪▪^,^31^]. The US NCCN currently endorses the use of genomic classifiers for clinical management and Decipher, in particular, has been given the highest level of evidence ratings. A number of studies have shown their use can influence clinician and patient perceptions of risk and subsequently, decisions on treatment [32].

While very promising, there are limitations to be considered in the evidence base for these tests in the localised disease setting. In most studies, genomic classifiers have shown improvement over older clinical models, for example the NCCN which we have discussed above as having inferior performance [8]. As an example, the AUC improvements when genomic classifiers are added to NCCN for prostate cancer mortality have been in the 0.74–0.80 range, which is poorer or similar to the best modern clinical models alone [8,30^▪▪^,^33^]. Genomic classifiers have shown correlation with different intermediate outcomes including biochemical relapse and metastatic progression [29,30^▪▪^,^31^]. The consistency and incremental value in improving predictions however remains debated. Boyer *et al.*[30^▪▪^] in a comprehensive systematic review highlighted the very variable performance of genomic classifiers, inconsistent comparison arms and general low-level evidence grade. In head-to-head comparisons, the performance of each test also appears to differ depending on the outcome of interest. Lehto *et al.*[33] in men treated by radical prostatectomy found that Oncotype DX had the worst performance in predicting metastasis, while Prolaris was the worse for prostate cancer mortality. Results for predicting outcomes also differ from study to study. Cooperberg *et al.*[34] added genomic classifiers to the CAPRA classification and did not find improved prediction of prostate cancer mortality after radical prostatectomy (though it did help sub-categorise men in different clinical groups). In a follow-up study, while an improvement in mortality prediction was seen the effect was modest (AUC improvement of 0.04) [35]. In the active surveillance context, Vince *et al.*[36] found that using genomic classifier tests influenced the time men spent on surveillance, but there was no significant difference in re-biopsy grade re-classification between men with different genomic classifiers scores. In contrast, Press *et al*. [37] found that genomic classifiers did predict upgrading, but that this was confined to men with initial Grade Group 1 disease only; here again, the AUC improvement was modest (increase of 0.06). Perhaps the most useful role for genomic classifiers is to help guide modifications in disease treatments regimes [38]. Decipher, for example, has shown utility to predict the benefit from different durations of antiandrogen therapy in local disease treated by radiotherapy and in the postsurgery salvage setting [30^▪▪^,^38^,^39^].

The actual survival gains from changing a treatment decision or regime based on a genomic classifiers score remains uncertain. At present, there is a risk that use of genomic classifiers may change decisions on management only on a presumption of benefit [31,40]. The current era of trials testing treatment escalation/de-escalation based on genomic classifiers will be important; for example, the G-Major and G-Minor trials from the Michigan consortium, radiotherapy trials from the NRG (e.g. PREDICT-RT) and surgery trials like the GUNS study [38,41]. There also remain the question of how MRI-targeted biopsy as opposed to systematically derived biopsies may change genomic classifiers scores in localised disease. This is particularly relevant if genomic classifiers are to be profiled in biopsy samples as opposed to whole mount radical prostatectomy tissue. In one study, significant heterogeneity was found in the genomic classifiers scores from different biopsy cores even within MRI-targeted biopsies [42]. While MRI-directed biopsies in general derived the highest grade and genomic classifiers scores, this may again conversely result in a risk inflation of apparent as opposed to actual risk as discussed before [42,43^▪▪^].

## ARTIFICIAL INTELLIGENCE

The use of artificial intelligence in cancer has exploded in recent years. In prostate cancer, most applications have so far been to automate and increase efficiency for image reporting, radiomics and histopathology reporting [44^▪▪^,^45^,^46^]. Artificial intelligence models in prostate cancer prognosis are being explored but just like traditional statistical models they rely entirely on the quality of the data used and the outcomes that the models are trained against [47^▪▪^,^48^]. In clinical modelling for prognosis, they have not yet shown superiority or better performance [10].

Perhaps the most prominent at present is the Artera-AI tool, which has been recently endorsed by the NCCN (2024) for predictive and prognostic use in prostate cancer [4]. Artera-AI is a Multi Modal Artificial Intelligence (MMAI) system that incorporates clinical data and histological slide information to predict clinical outcomes. The unique variables in this system, in addition to clinical factors, is a fusion of digitised slide images (a so-called image quilt) [49]. In the key article describing the model, depending on whether the outcome was survival or metastasis, the improvements in AUC was between 0.02 and 0.14 over NCCN categories only [49]. In subsequent studies using this MMAI system, it has been demonstrated to better predict benefit from different durations of antiandrogen therapy and for sub-dividing men with high-risk disease by metastatic potential [50^▪^,^51^]. Based on these results and the fact that the studies included data from randomised trials, the NCCN endorsed the use of Artera-AI in prostate cancer management. More recently, this it has also been tested retrospectively in the SPARTAN trial of nonmetastatic castrate treated with Apalutamide [52]. In this study, the model was able to differentiate men at different risks of metastasis-free, second progression-free survival and overall survival using a dichotomous low or high MMAI score.

Like with imaging and genomic classifiers, key questions remain about the actual added incremental benefit that artificial intelligence based models will bring to clinical management particularly if compared with more modern clinical classification systems. Like genomic classifiers, the performance of tissue-based MMAI also depends on the tissue sampled for histology. Hence, how MRI/targeted derived biopsy information versus systematic biopsies (used in all trials testing MMAI studies so far) influence the scores outputted is unknown. Ultimately like other biomarkers, the key question will be how management would be changed based on its use.

## CONCLUSION

There are a plethora of new biomarker-based models seeking to improve, optimise or replace clinical models. There are currently many options available, but there needs to be caution in recommending their use. Most comparative studies overlook the fact that clinical models themselves have evolved over time. This is important, as clinical models are free, whereas every other modality will bring extra costs. It is also important to distinguish whether a biomarker model is for prognosis or predictive use and the lines between the two are often blurred in the literature. Using biomarker models without clear evidence of added prognostic value may result in over-treatment and undue anxiety especially in localised disease decision making [53]. Conversely, if used as predictive markers where treatment modulation is possible, it may help avoid undue side effects for some men while improving the likelihood of better disease control for others.

For any these assets to become the standard of care however, well designed prospective clinical trials are needed to answer the “what would you do differently” question and it is good to see that many are underway. Until then, it is imperative that users (clinicians and patients) balance the incremental value of a (costly) new biomarker against the performance of the latest clinical classifications and multivariate models when deciding on the value of use. This is especially important in the context of localised prostate cancer where often the most important question is, does a patient actually need any treatment?

## Acknowledgements

*Vincent J. Gnanapragasam acknowledges infrastructure support from the NIHR Cambridge Biomedical Research Centre (BRC-1215-20014). The views expressed are those of the authors and not necessarily those of the NIHR or the Department of Health and Social Care.*

*V.G. analysed and reviewed articles and wrote the articles.*

### Financial support and sponsorship

*None.*

### Conflicts of interest

*There are no conflicts of interest.*

## REFERENCES AND RECOMMENDED READING

Papers of particular interest, published within the annual period of review, have been highlighted as:

▪ of special interest
▪▪ of outstanding interest
